# The war on marijuana: The transformation of the war on drugs in the 1990s

**DOI:** 10.1186/1477-7517-3-6

**Published:** 2006-02-09

**Authors:** Ryan S King, Marc Mauer

**Affiliations:** 1 The Sentencing Project, 514 10^th ^Street, NW, Washington, DC 20004, USA.

## Abstract

**Background:**

As the "war on drugs" enters the latter half of its third decade since being forged into the American lexicon by President Ronald Reagan, the public has grown more skeptical of the current strategy and has proven to be receptive to a broader consideration of alternatives to incarceration. This has been the case most notably with marijuana offenses, where the policy discussion has shifted in some localities to one of decriminalization or de-prioritizing law enforcement resources dedicated to pursuing possession offenses. Despite the increased profile surrounding marijuana policy in recent years, there remains a significant degree of misunderstanding regarding the current strategy, both in terms of how resources are being allocated and to what eventual gain.

**Methods:**

Previous studies have analyzed drug offenses as a general category, but there has yet to be a single study that has focused specifically on marijuana offenders at all stages of the system. This report analyzes multiple sources of data for the period 1990–2002 from each of the critical points in the criminal justice system, from arrest through court processing and into the correctional system, to create an overall portrait of this country's strategy in dealing with marijuana use.

**Results:**

The study found that since 1990, the primary focus of the war on drugs has shifted to low-level marijuana offenses. During the study period, 82% of the increase in drug arrests nationally (450,000) was for marijuana offenses, and virtually all of that increase was in possession offenses. Of the nearly 700,000 arrests in 2002, 88% were for possession. Only 1 in 18 of these arrests results in a felony conviction, with the rest either being dismissed or adjudicated as a misdemeanor, meaning that a substantial amount of resources, roughly $4 billion per year for marijuana alone, is being dedicated to minor offenses.

**Conclusion:**

The results of this study suggest that law enforcement resources are not being effectively allocated to offenses which are most costly to society. The financial and personnel investment in marijuana offenses, at all points in the criminal justice system, diverts funds away from other crime types, thereby representing a questionable policy choice.

## 

The War on Marijuana: The Transformation of the War on Drugs in the 1990s

*Federal law enforcement is targeted effectively at convicting major drug traffickers and punishing them with longer lockups in prison*. [1]

-John Ashcroft, Former United States Attorney General

## Background

Despite decades of discussion and intense media coverage, there remains considerable confusion regarding how the criminal justice system treats marijuana offenders. This misunderstanding has catalyzed a contentious debate that has been characterized by disagreements about the appropriate legal status of marijuana, the suitable level of punishment, and the most effective distribution of institutional resources to address marijuana use. This has been coupled with a fundamental difference of opinion about the true dangers that marijuana use poses to American society. In light of international developments in which a number of countries have reduced punishment for marijuana use, as well as the growth in the domestic decriminalization movement culminating in local ballot initiatives and proposals to amend state law, the struggle over the appropriate criminal justice response to marijuana has become a key policy concern.

Drug war advocates such as John Walters and former Attorney General John Ashcroft have frequently remarked that the current criminal justice approach to drug abuse represents an efficient use of resources. Walters, the head of the Office of National Drug Control Policy, has lamented that persons who claim that prisons are full of low-level drug offenders are incorrect and have misinformed the debate on drug policy [2].

In order to provide a framework for assessing the role of marijuana enforcement in the criminal justice system, we have conducted a national analysis of marijuana offenders for the period of 1990 to 2002. This includes an assessment of trends in arrest, sentencing, and incarceration, along with an evaluation of the impact of these developments on marijuana price and availability, and the use of crime control resources. Our analysis indicates that the "war on drugs" in the 1990s was, essentially, a "war on marijuana."

Key findings include:

▪ Of the 450,000 increase in drug arrests during the period 1990–2002, 82% of the growth was for marijuana, and 79% was for marijuana possession alone;

▪ Marijuana arrests now constitute nearly half (45%) of the 1.5 million drug arrests annually;

▪ Few marijuana arrests are for serious offending: of the 734,000 marijuana arrests in 2000, only 41,000 (6%) resulted in a felony conviction;

▪ Marijuana arrests increased by 113% between 1990 and 2002, while overall arrests decreased by 3%;

▪ New York City experienced an 882% growth in marijuana arrests, including an increase of 2,461% for possession offenses;

▪ African Americans are disproportionately affected by marijuana arrests, representing 14% of marijuana users in the general population, but 30% of arrests;

▪ One-third of persons convicted for a marijuana felony in state court are sentenced to prison;

▪ One in four persons in prison for a marijuana offense – an estimated 6,600 persons – can be classified as a low-level offender;

▪ An estimated $4 billion is spent annually on the arrest, prosecution and incarceration of marijuana offenders.

The findings in this report call for a national discussion regarding the zealous prosecution of marijuana use and its consequences for allocation of criminal justice resources and public safety. Law enforcement has focused disproportionately on low-level possession charges as a result of the nation's lack of a thoughtful strategy about how best to address the consequences of marijuana use. Consequently, police spend a significant amount of time arresting marijuana users, many of whom do not merit being charged in court. This diverts efforts away from more significant criminal activity while having no appreciable impact on marijuana cost, availability, or use. As state and federal resources become more limited, a rational consideration of the most efficient way to address marijuana use is critical; this discussion should take place outside the realm of political rhetoric. The findings in this study can inform that conversation with sound, empirical analysis of more than a decade's worth of data on the criminal justice system's treatment of marijuana offenders.

### Law enforcement and marijuana

**Figure 1:**
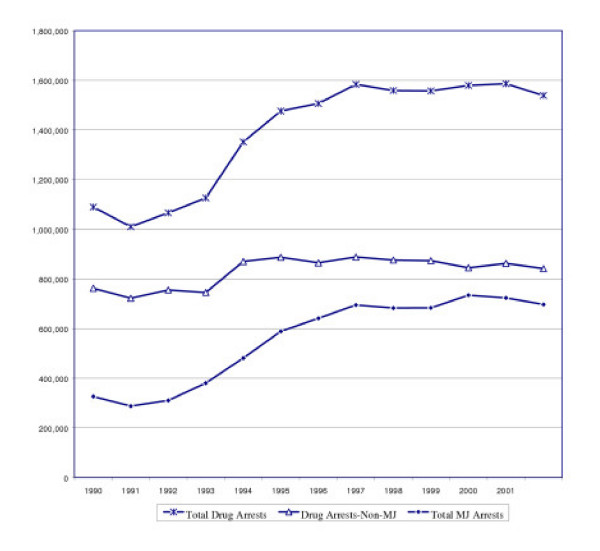
Drug Arrest Trends – 1990 to 2002.

As seen in Figure 1, from 1990 to 2002, drug arrests nationally increased by 41%, from 1,089,500 to 1,538,800. During this time, the total number of marijuana arrests more than doubled from 327,000 to 697,000, an increase of 113%. All non-marijuana drug arrests increased by only 10%. The percentage of arrests for all offenses comprised of marijuana more than doubled from 2.3% in 1990 to 5.1% in 2002.

This significant expansion of the drug war was fueled almost entirely by a focus on marijuana. Of the 450,000 increase in arrests for drugs, 82.4% was solely from marijuana arrests, and 78.7% from marijuana possession arrests.

Since 1990, there have been 6.2 million arrests for marijuana possession and an additional 1 million for marijuana trafficking. As of 2002, marijuana arrests comprised 45% of all drug arrests, and of these, possession arrests constituted 88% of all marijuana arrests. While marijuana *trafficking *arrests declined as a proportion of all drug arrests during this period (from 6.1% in 1990 to 5.4% in 2002), the proportion for marijuana *possession *increased by two-thirds (24% in 1990 to 40% in 2002).

**Figure 3:**
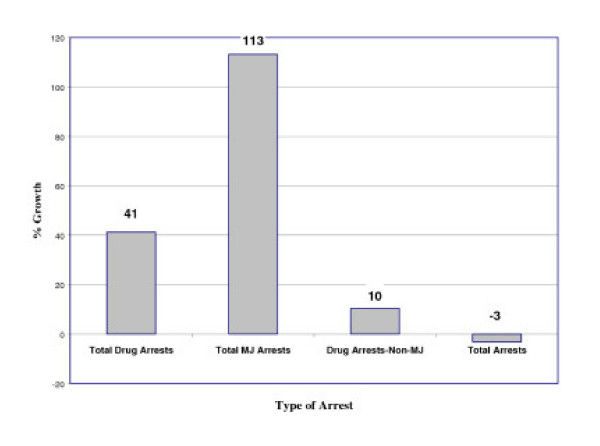
Growth in Arrests – 1990 to 2002.

As seen in Figure 3, overall arrests declined by 3% from 1990 to 2002, while marijuana arrests rose by 113%. The incongruent arrest patterns between marijuana and other criminal offenses require further analysis to understand the trends at work. Overall Index I crimes, defined as the most serious and costly to society, dropped by 24% during this period, a time when the United States was experiencing the lowest crime rates since the 1970s [3].

From a policy perspective, for this growth to be tenable, one must assume that marijuana use and marijuana market trends ran counter to all national crime trends, including patterns in overall drug arrests. As this is rather unlikely, this growth is probably better understood as the result of selective enforcement decisions. There is no indication from national drug survey data that a dramatic decrease in the use of other drugs led to law enforcement agencies shifting resources to marijuana [4]. Indeed, there was a slight *increase *in the use of all illicit drugs by adult users between 1992 and 2001 (5.9% to 6.6%) [5]. Over that same period, emergency room admissions for heroin continued to increase [6]. Thus, there are no explicit indications of dramatic shifts in drug use that might explain the law enforcement trend toward marijuana enforcement in the 1990s.

**Figure 4:**
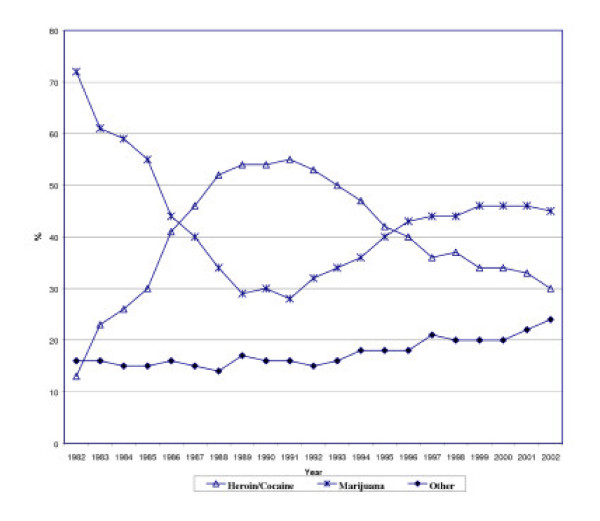
Trends in Drug Enforcement – 1982 to 2002*. * = Chart adapted from data in Pastore, AL and Maguire, K (Eds.): **Sourcebook of Criminal Justice Statistics 2001**. United States Department of Justice, Bureau of Justice Statistics, Washington, DC: USGPO, 2002. (Table 4.29). Additional data obtained from **Crime in the United States, 2001 **(Table 4.1) and **Crime in the United States, 2002 **(Table 4.1). Washington, DC: Federal Bureau of Investigation.

An examination of historical drug arrest patterns illustrates the role of policy decisions in shaping the trends of the 1990s. As seen in Figure 4, in 1982, marijuana comprised 72% of all drug arrests. At that point, the "war on drugs" began in earnest and there was a shift in the distribution of arrests for drug abuse violations. By 1992, marijuana arrests made up only 28% of all drug arrests. During that same period, the proportion of cocaine and heroin arrests increased from 13% to 55%.

However, over the course of the 1990s a tangible shift toward arrest patterns of the early 1980s began to reemerge. Law enforcement agencies arrested fewer people for cocaine and heroin offenses and began to arrest more people for marijuana possession and sale. By 1996, marijuana had once again surpassed heroin and cocaine as the primary drug of arrest, a gap which has widened since then. Early pursuit of the "war on drugs" targeted heroin and cocaine (drugs deemed to be *hard, costly*, or *dangerous*), but the current manifestation of the drug war, from the law enforcement perspective, is targeted disproportionately at marijuana use.

### Impact on marijuana use

What impact has the practice of increased arrest for marijuana offenses had on rates of use, ease of purchase, and price? Higher trafficking arrests theoretically should reduce supply and increase marijuana costs, and an increase in possession arrests should, if general deterrence theory applies, reduce use through heightened probability of apprehension. However, since 1992, real price has fallen by 16% while potency has increased by 53% [7]. From 1990 to 2002, daily use of marijuana by high school seniors nearly tripled from 2.2% to 6%. Notably, the current 6% level is the same as the level in 1975 [8]. One study suggests that the rapid increase in low-level arrests, many of which result in dismissals or misdemeanor convictions, reinforces a perception that a person can "get away with it" [9]. Consequently, the frequent use of marijuana arrests provides little of the deterrent effect necessary to put pressure on the market exchange.

Thus, after 30 years of aggressively pursuing marijuana, arrests have grown at a rapid rate while use patterns fluctuate, but remain near the same level. In 1990, 84.4% of high-school seniors responded that it was *fairly easy *or *very easy *to get marijuana. Despite a record number of arrests, this figure actually *increased *slightly over the 12-year period of the study to 87.2%, near 1975 levels [10].

**Figure 5:**
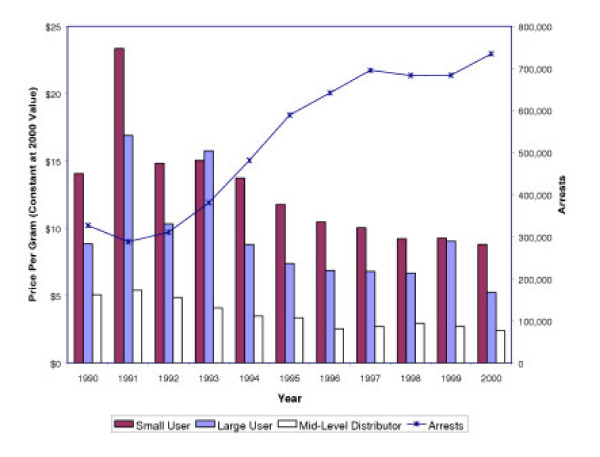
Trends in Marijuana Pricing and Law Enforcement – 1990 to 2000.

The continued ease with which users obtain marijuana calls into question the wisdom of the national investment of increased law enforcement targeting marijuana users. Recent research suggests that raising the price of marijuana has a significant impact on its use; however, law enforcement has not succeeded in raising prices [11]. Figure 5 illustrates the growth of marijuana arrests plotted alongside the average annual cost for marijuana by the gram [12]. Marijuana prices are measured at 2000 values to allow for comparison. In all three categories of marijuana distribution, costs have dropped consistently since 1991.

Despite a 113% increase in marijuana arrests, almost exclusively for possession, marijuana costs have decreased, and purity increased as have use and perceived availability. If increased law enforcement and an expanded use of arrests were successful in restricting the supply of marijuana, then an increase in its price would be expected. Instead, marijuana prices fell continuously during the 1990s even as marijuana arrests reached unprecedented levels. This trend suggests that the growth in marijuana arrests in the 1990s has had no measurable impact on price, access, or availability.

The drug war has been predicated on arresting high-ranking narco-traffickers, as the opening quote by former Attorney General Ashcroft illustrates. However, the data indicate that this has not been the practice. During the 1990s the focus of law enforcement has been on low-level marijuana offenders. In fact, some law enforcement officials acknowledge that they target low-level offenders as part of a larger strategy known as "quality of life," or order maintenance, policing. This approach emphasizes the use of police officers to stop and frisk pedestrians under the assumption that such encounters will deter people from carrying contraband. In practice, this approach often targets low-level offenses as a means of identifying more substantial criminal behavior. Former New York City Police Commissioner Howard Safir remarked, in defense of this strategy, " [o]ur plan is to attack it on all levels. We're not just going after the major traffickers; we're gonna harass the little guys on a daily basis" [13].

This approach has had a disproportionate impact on the African American community. According to data from the National Survey on Drug Use and Health for 2002, approximately 74% of regular marijuana users (those who have used within the past month) are non-Hispanic whites and 14% are black, rates that are similar to lifetime use patterns as well (76% white and 11% black) [14]. But these figures contrast sharply with arrest rates. While blacks make up approximately 14% of marijuana users in the general population, they are 30% of those arrested for marijuana violations.

Enforcement policy decisions are one potential explanatory factor for the disparity in arrest by race. A Maryland study on marijuana enforcement observed that police officers knew where to go if they wished to make an easy drug arrest, and suggested that they could do so whenever they wished in certain neighborhoods [15]. These neighborhoods are those where drug use and selling is most likely to be in public spaces, allowing for easy apprehension. Research by criminologist Alfred Blumstein supports this point, observing that disproportionate arrest rates are due to "a more dense police presence where blacks reside" [16].

### The cost to law enforcement

The growth of marijuana arrests results in substantial costs for law enforcement. Since 1991, the domestic law enforcement component of the federal drug control budget has increased from $4.6 billion (or 42% of the federal drug control budget) to $9.5 billion (or 51% of the federal drug control budget) in 2002 [17]. This increase of $4.9 billion (107%) has occurred during a period when most of the growth in drug arrests has been for marijuana.

Of the total law enforcement budget for 2001 of $72.4 billion, we estimate that $2.1 billion, or 2.9% of the entire law enforcement budget nationally, is spent on marijuana arrests. Of this, approximately $430 million is spent on marijuana trafficking and $1.7 billion on marijuana possession arrests [18].

### Law enforcement resource allocation

In addition to cost, a significant consequence of these tactics includes reduced law enforcement attention to other criminal behavior. Law enforcement resources come from a finite pool of funding in the general revenue fund. It is the responsibility of the legislature to determine how these resources will be allocated (law enforcement, corrections, education, roads, etc.). If the role of law enforcement is to be expanded, there are three options available to accomplish this: 1) increase the size of the common pool (raise taxes); 2) alter the distribution within the common pool (draw monies away from a different program and direct additional funding towards law enforcement); or 3) alter the approach of law enforcement patterns (practice selective enforcement of offenses).

Economists Rasmussen and Benson believe that the latter is the most likely solution. " [I]ndividual police officers and police departments as a whole must decide which laws to attempt to enforce and how rigorously" [19]. Increased resources directed towards a specific type of offense, such as drugs, lead inevitably to a decrease in resources dedicated to another offense. Law enforcement resource allocation is a zero-sum game, and any difference in appropriation is likely to manifest itself in delayed response times. "As drug crimes receive more attention from police ... the queues for other offenses must move slower as fewer resources are allocated to them" [20]. This is of particular concern in light of recent developments which indicate that many cities and municipalities are losing police officers in response to budgetary constraints [21].

Benson, Rasmussen and Kim find support for this hypothesis. Looking at data from Florida, they conclude that every additional drug arrest leads to an increase of 0.7 Index (serious) crimes [22]. The authors surmise that increased law enforcement of drug offenses has a dual effect: it directs resources away from the pursuit of Index crimes, and it may drive potential economically motivated drug offenders into non-drug crimes (some of which may be more dangerous or costly to society) where law enforcement attention is not as greatly concentrated. A quasi-replication of that study using more recent data from Florida found that a one percent increase in drug arrests would lead to a .18% increase in Index crime [23]. The impact of the war on drugs on the enforcement of other crimes has been demonstrated elsewhere. A study of New York State law enforcement data found that an increase in drug arrests for sale and possession led to a significant increase in assaults, robberies, burglaries, and larcenies [24]. Moreover, a 10% increase in marijuana sale arrests led to 880 additional larcenies statewide [25].

The shift in the 1990s towards more aggressive policing of marijuana may have siphoned law enforcement resources away from certain Index crimes. Rasmussen and Benson suggest that not only is this a possible scenario, but that there are institutional incentives in place that encourage the pursuit of drug crimes. Civil asset forfeiture, which permits law enforcement agencies to seize all or a portion of property obtained during a drug investigation, creates an incentive for administrators to dedicate more resources to drug enforcement. " [L]aw enforcement agencies focus resources on enforcement of drug laws because of the financial gains for the agencies arising from forfeitures" [26]. Indeed, a recent analysis of arrest patterns in police departments that are permitted to retain a portion of seized assets discovered that this policy resulted in an increase of drug arrests by 18%, and drug arrests as a portion of all arrests by 20% [27].

### Marijuana enforcement at the local level

The geographical variation in marijuana arrest patterns at the local level illustrates the critical role of discretion in defining a law enforcement agency's policy. Table 1 provides the number of arrests in 1990 and 2002 in the nation's ten largest counties as well as ten other large counties chosen for their geographic distribution, taken from the Uniform Crime Report [28].

**Table 1:** Marijuana Arrests-Large U.S. Counties – 1990 and 2002****

**County**	**1990 Sale**	**2002 Sale**	**% Growth**	**1990 Poss.**	**2002 Poss.**	**% Growth**	**1990 Total**	**2002 Total**	**% Growth**
*Los Angeles (CA)*	6,708	2,868	-57	5,638	12,929	129	12,346	15,797	28
Cook (IL)*	8,974	N/A	N/A	1,597	N/A	N/A	10,571	N/A	N/A
*Harris (TX)*	68	38	-44	1,593	7,174	349	1,661	7,212	334
*Maricopa (AZ)*	563	462	-18	3,529	6,194	76	4, s092	6,656	63
*Orange (CA)*	636	579	-9	3,128	6,466	107	3,764	7,045	87
*San Diego (CA)*	1,588	756	-52	3,162	4,950	57	4,750	5,706	20
Miami-Dade (FL)*	1,279	N/A	N/A	3,926	N/A	N/A	5,205	N/A	N/A
*Dallas (TX)*	174	260	49	2,483	2,992	20	2,657	3,252	22
*Wayne (MI)*	182	223	23	1,009	2,357	134	1,191	2,580	117
*King (WA)*	94	187	99	639	3,608	465	733	3,795	418

Philadelphia (PA)	468	2,449	423	358	3,774	954	826	6,223	653
Middlesex (MA)	93	233	151	676	1,274	88	769	1,507	96
Cuyahoga (OH)	161	141	-12	732	1,032	41	893	1,173	31
Clark (NV)	9	560	6,122	98	3,472	3,443	107	4,032	3,668
Hennepin (MN)	25	467	1768	739	1,184	60	764	1,651	116
St. Louis (MO)	106	126	19	617	1,625	163	723	1,751	142
Fairfax (VA)	53	18	-66	383	258	-33	436	276	-37
Milwaukee (WI)	363	840	131	1,542	2,228	44	1,905	3,068	61
Shelby (TN)	34	584	1,618	91	1,790	1,867	125	2,374	1,799
Fulton (GA)	314	776	147	2,750	3,757	37	3,064	4,533	48

**** – Cook County, Illinois and Miami-Dade County, Florida did not provide marijuana arrest statistics to the Uniform Crime Report in 2002.

Several key findings can be identified from these figures:

• Every major county except for Fairfax, Virginia experienced an increase in marijuana arrests between 1990 and 2002;

• The growth rates in marijuana arrests in the top 10 counties ranged from 20% (San Diego, CA) to 418% (King, WA);

• In 1990, 11 of the 20 counties in our sample had more than 1,000 marijuana arrests; in 2002, all but one had more than 1,000 marijuana arrests.

A number of counties had very few arrests in 1990, so the growth these counties experienced produced astronomical increases. Eight counties more than doubled their marijuana arrests between 1990 and 2002, with some increasing five- and ten-fold. In addition to the near uniform patterns of growth, the other noteworthy trend was the consistent growth in arrests for possession. In nearly every county in the sample, the growth rate for possession arrests far exceeded that for sales or manufacturing. Despite the similarities, there are variations in the degree of growth, and there are also a number of counties that experienced a decline in some types of marijuana arrests. In the ten most populous counties, the growth in arrests ranged from 20% (San Diego) to 418% (King, WA). This variation was affected by the size of the county and the degree to which each had been pursuing marijuana violations in 1990 versus 2002. It also underscores the importance of individual policymakers and practitioners making decisions that shift the emphasis in enforcement policy. Short of a localized, rapid increase in marijuana sales and use, for a county to experience the size of growth witnessed in Clark (Nevada), Shelby (Tennessee), or most of the counties in this sample, a tangible modification of marijuana arrest policies is the most likely cause. As a means of demonstrating this point, we examine the trends in marijuana arrest patterns in New York City and discuss the impact of contemporaneous political developments.

### Marijuana at the city level

Examining city level arrest patterns is an instructive approach to provide context to national level trends. In the case of New York City, the 1990s represented a profound shift in policing strategy that resulted in an exponential growth in marijuana arrests. Although the experience in New York City may not be representative of developments across the country, it is an example of the ways in which the decisions of local officials played a role in the national increase in marijuana arrests.

Prior to the election of Mayor Rudolph Giuliani in 1994, the New York City Police Department applied a low-key approach towards marijuana use and distribution. Marijuana offenses were usually treated with a fine issued in the form of a citation, and in many cases individuals were not required to report to court, but were permitted to handle the ticket in the same fashion as a traffic summons. The election of Mayor Giuliani, with a promise of addressing "quality of life" issues [29] in New York City, and the subsequent appointment of William Bratton as police commissioner, ushered in a new era of policing in the city.

Shifting away from the "community policing" model of the previous Dinkins administration, Commissioner Bratton implemented a strategy of "zero tolerance" policing. Grounded in the philosophy of "broken windows," zero tolerance policing is characterized by aggressive policing of traditionally ignored, minor offenses. If left unchecked, according to supporters of this approach, this allows an element of criminality to take root in a community, leading to more serious criminal behavior. New York police officers increased their use of stop-and-frisk searches in an effort to crack down on public nuisance offenses. In 1997, a New York Police Department unit of 433 officers performed stop-and-frisk searches on about 45,000 people [30]. In the process of targeting public-nuisance issues, the Department also began a revitalized pursuit of marijuana offenders.

The new policing strategy was crafted to target violations of public order, and to increase the overall risk of apprehension for all offenses. By casting as wide a net as possible, the police sought to increase the likelihood of apprehending more substantial criminal offenders. Supporters of this approach contend that increasing the probability of arrest provides a deterrent effect to persons carrying weapons or illegal drugs on the street.

The way in which law enforcement implements this policy is through increased use of the "stop-and-frisk" technique. "A 'stop' intervention provides an occasion for the police to have contact with persons presumably involved in low-level criminality" without meeting the evidentiary burden necessary to make an arrest, while providing a pretense for further investigation [31]. One New York Police official described the approach: " [y]our open beer lets me check your ID. Now I can radio the precinct for outstanding warrants or parole violations. Maybe I bump against that bulge in your belt; with probable cause, I can frisk you" [32]. A legal "stop" may result in nothing more than a verification that an individual was engaged in legal behavior. But it also may result in an arrest for a low-level offense, or, and this is the reason cited by supporters, it may result in the identification of more serious criminal behavior.

Such an approach is more intrusive than conventional policing and increases the likelihood that persons will face arrest for low-level violations, such as marijuana use, that may have been ignored in the past. Moreover, opponents contend that this aggressive approach results in racially discriminatory arrest patterns and is tantamount to police harassment. The growth in the use of the "stop-and-frisk" technique in New York City in the 1990s unquestionably was partly responsible for the increase in the number of low-level marijuana arrests during that period. The discretion that police officers have in initiating a stop is evidenced by the fact that less than one-third of all encounters are the result of a person meeting the description of a suspect for a crime [33]. An analysis of more than 175,000 "stop-and-frisk" encounters during a 15-month period in 1998 and 1999 indicated that African Americans and Hispanics were substantially more likely to be stopped by the police, and this difference could not be explained by legal factors such as criminality or overall crime rates [34].

**Figure 7:**
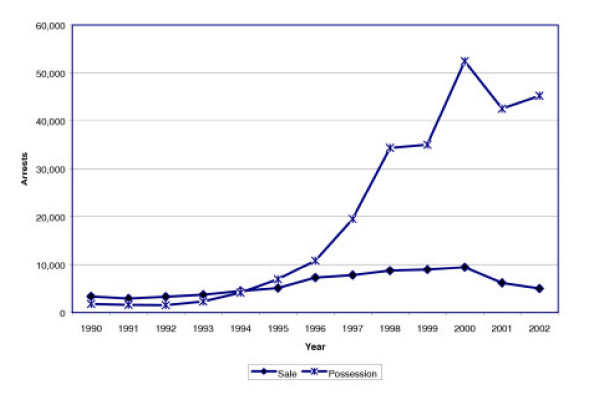
Marijuana Arrests in New York City – 1990 to 2002***. *** = Data from the New York State Division of Criminal Justice Services, Computerized Criminal History System.

As Figure 7 illustrates, the increased use of "stop-and-frisk" encounters produced a dramatic rise in marijuana arrests, most of which focused on use rather than trafficking. In 1990, there were 5,116 arrests for marijuana offenses, of which 34.5% (1,766) were for possession. By 2002, marijuana arrests had increased by 882%, totaling more than 50,000. Of those, 90% (45,227) were for possession offenses, representing an increase of more than 2400%. In contrast, total arrests for all offenses were up only 8%. Arrests for violent crimes dropped 33% and felony drug crimes dropped 39%. Not surprisingly, misdemeanor drug arrests increased by 143%, driven by growth in marijuana arrests. If the growth in marijuana possession arrests is removed from the equation, overall drug arrests in New York City would have dropped by nearly 15,000. This trend should come as little surprise, as some experts suggest a byproduct of order maintenance policing will be an increase in arrests for low-level offenses [35].

Nationally, the proportion of marijuana arrests from New York City also grew exponentially. In 1990, 1.6% of all marijuana arrests nationally occurred in New York City; that figure more than quadrupled to 7.2% by 2002. The proportion of possession arrests from New York City grew ten-fold, from 0.7% to 7.4% of national arrests. This translates into more than 12% of the growth in national marijuana arrests between 1990 and 2002. While New York City represents approximately 3% of the nation's population and 2.1% of the nation's total arrests, more than 7% of all marijuana arrests in the entire country in 2002 occurred in New York City. These figures indicate a citywide policy that, in the process of executing a zero-tolerance policing model, has demonstrably shifted towards the targeting of marijuana users for arrest.

### Alternative enforcement strategies: domestic and international

Although not all large American cities were experiencing as significant an upward turn in marijuana arrests as in New York City, the country was moving in that direction on a national scale. Domestically the rapid growth in marijuana arrests has led to a number of situations in which local officials have succumbed to the overwhelming increase in workload and have responded by demanding a change in practice. In 2000, local district attorneys in Texas declined to accept low-level drug prosecutions from federal agencies because they could not keep up with the number of arrests [36]. One district attorney noted, "We wanted to do our share of fighting the war on drugs. But now it's too much" [37].

In Syracuse, the proportion of resources dedicated to drug enforcement raised concern with the city's auditor. The auditor's report to the mayor noted that drug-related arrests "exceeded arrests for assaults, disturbances, and larcenies combined," and that arrests for marijuana comprised nearly one-third of all drug arrests [38]. City Auditor Minchin G. Lewis expressed concern that the department's emphasis on pursuing drug use had unintended consequences for the community and was an inefficient use of law enforcement resources. Lewis recommended that the Common Council of Syracuse conduct a survey of neighborhood residents and identify the level to which residents are concerned with drug related incidents. "Devoting so many taxpayer resources in an effort to eliminate the distribution of drugs could be a significant misappropriation of resources if the primary concern is not drug use" [39].

In Chicago, similar concerns have been raised, this time in a report submitted by a city police sergeant [40]. Sergeant Thomas Donegan noted that the vast majority (over 90%) of marijuana arrests in Chicago were dismissed or dropped, leading him to question why law enforcement agents were dedicating significant resources to pursue marijuana when approximately nine of ten cases will not result in a conviction. Donegan recommended the use of fines rather than arrest for marijuana use, a proposal endorsed by Chicago Mayor Richard M. Daley.

These concerns of practitioners are translating into reform measures at the ballot box. In the late 1990s, Arizona passed Proposition 200, which among other reforms, explicitly noted that drug use was to be treated as a health problem, with monies dedicated to treatment and education rather than incarceration. More recently, Proposition 36 in California created a protocol to divert low-level drug offenders into treatment rather than prison. In Seattle, the passage of Initiative 75 reduced marijuana for adult personal use to the lowest law enforcement priority, estimated to be saving tens of thousands of dollars in state prison and local jail costs. In addition to the numerous state initiatives in the past few years permitting the use of marijuana for medical purposes, there have also been efforts to reform the response to recreational use.

In the 2004 election, a number of state and local initiatives addressed the need for change in the manner in which law enforcement treats marijuana use [41]. In Oakland, California, Measure Z, which was passed with 64% of the vote, will "make investigation, arrest, prosecution, and imprisonment for private adult cannabis offenses the lowest law enforcement priority ..." In Columbia, Missouri, 61% of voters supported reforms to the current criminal code that will reduce the punishment for possession to a $250 fine and keep marijuana cases in municipal court. This legislation resembles approaches employed in Ann Arbor, Michigan and Madison, Wisconsin.

In addition to these domestic changes, there have also been similar developments abroad. In May of 2004, Russia decriminalized the possession of small amounts of narcotics, including marijuana. Canada continues to debate decriminalization of marijuana, but in June of 2003, Toronto Police Chief Julian Fantino declared that he had instructed his officers to discontinue making arrests for simple possession of marijuana [42]. In response to the developments in Toronto, the head of the provincial association of police chiefs suggested to all law enforcement in Ontario to "use discretion in situations that involve the simple possession of marijuana" [43].

England has also been experimenting with decriminalization. In August of 2001, the police commissioner in Brixton announced that the police would no longer be making arrests for personal possession of marijuana, as a result of the police and courts being overrun with marijuana related arrests. In Brixton, about 90% of all drug arrests are for possession and about 75% of those possession arrests are for marijuana [44]. By the summer of 2002, it was announced that all of Britain would be decriminalizing personal amounts of marijuana [45]. Former Home Secretary David Blunkett stressed that the decriminalization of marijuana did not equate to decriminalization of all behaviors related to the use of marijuana. "Where cannabis possession is linked to aggravated behavior that threatens public order, the police will retain the power of arrest," while noting that this policy will permit Britain to "concentrate [our] efforts on the drugs that cause the most harm ..." [46].

The common thread in these developments has been a response by officials to the significant amount of time and resources that law enforcement officials dedicate to the pursuit of marijuana offenses. Dissatisfaction with the pursuit of the war on drugs has led a number of the nation's leading policing administrators to call for reform. A 2004 survey of 300 chiefs of police indicated that two-thirds of respondents believed that the response by law enforcement to drug use and sale had been unsuccessful [47]. Three-quarters of police chiefs believe that the resource gap for the enforcement of drug laws is more significant than with any other criminal or public safety requirements [48]. For these reasons, 84% of chiefs of police felt that there need to be changes implemented in the way drug laws are enforced [49].

### Assessing the increase in marijuana arrests

During the period after the beginning of the modern "drug war," a measurable shift in drug enforcement strategy could be identified. As seen previously in Figure 4, at the beginning of the 1980s, nearly three-quarters of all drug arrests were for marijuana; by the end of the decade that percentage had dropped to one-third. But by the early 1990s, drug enforcement relative to other arrests began to diminish. Rasmussen and Benson observe that between 1989 and 1990, the ratio of drug arrests to Index I crime arrests dropped by 24% [50]. This led the authors to conclude that the "drug war" was winding down relative to general law enforcement trends, or at least shifting the way that it was being pursued.

With the benefit of hindsight, fourteen years later it is clear that the drug war did not wind down. However, a closer examination of the growth in drug arrests indicates that there was a kernel of truth to the Rasmussen and Benson prediction about the abatement of drug enforcement. The 1990s ushered in a fundamental change in the way the drug war was pursued. As the crack cocaine market began to constrict in urban areas and the use of cocaine, heroin, and other substances remained flat, the category of primary drug abuse violation arrests skewed significantly towards marijuana, and particularly possession. Of the nearly one-half million increase in drug abuse violations between 1990 and 2002, 79% was for marijuana possession.

The precipitous growth of marijuana possession arrests is striking in the context of declining overall crime rates and stability in other drug abuse violations, as well as general use patterns nationally. Moreover, the growth in marijuana possession arrests beginning in the early 1990s was incongruous with the initial justification of the war on drugs in the 1980s, which stressed cocaine and heroin as targeted substances.

Marijuana arrests may have increased during the 1990s as a function of structural changes in criminal behavior and law enforcement strategies that increased the likelihood of marijuana arrests within the pre-existing routines of law enforcement officers. By the early 1990s, drug abuse arrests were leveling off and overall crime rates were decreasing as well. Beginning in 1992, violent and property crime would begin a sustained reduction for the longest duration in 30 years. During that same period, President Clinton was spearheading a movement to place 100,000 more police officers on the streets as a means of combating crime. The decade of the 1990s was characterized by these two trends: rapidly decreasing violent and property crime (beginning prior to the implementation of the national growth in police officers) and a rapid increase in law enforcement manpower.

These two trends may have combined to increase the probability of arrest for marijuana offenses. Law enforcement agencies prioritize their labor allocation; if serious crime is an entrenched problem in a given area, minor violations are likely to receive less attention. However, as the degree of serious crime diminishes, law enforcement agents are likely to turn their attention to nuisance crimes such as prostitution and petty drug crimes. The growth in stop-and-frisk searches in New York City is an example of this shift in practice. Therefore, as violent and property crime dropped through the 1990s, the growing ranks of law enforcement officers were more likely to pay attention to minor crimes such as marijuana possession, which would drive up arrest rates [51].

There has been little study of the method in which law enforcement agents respond to marijuana possession, but a Maryland study is instructive regarding the manner in which many marijuana arrests occur [52]. The authors of this study found that the rise in marijuana arrests during the 1990s in Maryland was not the result of a conscious policy shift, as in New York City; rather, it was the result of traffic stops and patrols in neighborhoods that the officers knew were "drug hot spots" [53]. The authors concluded that " [m]any of these arrests are not targeted at marijuana possession itself but are incidental to traffic stops, drug enforcement more generally, disorderly conduct and other patrol activities" [54]. They pointed out that general use patterns of marijuana in different Maryland counties was not a predictor of marijuana arrests.

The results from Maryland suggest that the growth in marijuana arrests was not the result of an upper-level directive; rather, it may simply be the product of an aggregation of law enforcement officers making more arrests while in the process of their daily patrol routine.

In the case of New York City, it is apparent that the growth in marijuana arrests was the result of the use of zero tolerance policing, and particularly, an increase in "stop-and-frisk" encounters. This approach primarily resulted in possession arrests and, as we will demonstrate in the next section, most of these were dismissed. Given the substantial law enforcement investment in such cases, the question is whether using stops for low-level offenses as a pretense for detecting more serious criminality is an effective approach to public safety. It clearly is not the most efficient, as evidenced in an Attorney General report concluding that only one out of every nine stops resulted in an arrest for any type of crime [55]. While there is no definitive analysis of the New York crime decline to date, most observers believe that the interplay of a number of variables was responsible. These include solid economic development, a dwindling crack cocaine market, demographic changes, and sophisticated computer technology that increased police response times [56].

### Marijuana and the court system

Given the dramatic growth in marijuana arrests, it is instructive to examine how these cases have been handled by the court system. The primary source for national level sentencing data is the *National Judicial Reporting Program*, which issues a biennial survey of felony sentences in state courts [57]. We collected NJRP data from 1990 and 2000, analyzing the processing of marijuana offenders in the state court system [58]. Perhaps surprisingly considering the growth in the arrest rate, state court systems did not experience any rapid increase in marijuana offenders being sentenced for a felony offense. The proportion of all persons sentenced for a marijuana felony in state courts in 2000 was 3.6%, which is 39% higher than the proportion in 1990 (2.6%), but far below the 113% growth in arrests during this period. The key findings of the court analysis are the following:

#### • System dismisses large number of arrestees, likely misdemeanors

The state sentencing figures in 2000 indicate a similar pattern as in 1990, suggesting that pre-trial dismissals and the fact that most arrests were for low-level misdemeanors dramatically mediated the shift in law enforcement treatment of marijuana over the decade. For example, in 2000 there were 734,000 marijuana arrests and approximately 41,000 felony convictions in state and federal courts [59]. Thus, only 1 of every 18 arrests results in some type of felony sentence.

Considering the significant growth in arrests during this period and the relative stasis in felony sentences for marijuana in the state court system, it is apparent that the vast majority of the more than 700,000 arrests for marijuana in 2000 are for misdemeanors, or are dismissed for one or more of a variety of reasons.

#### • Of those convicted of a felony, one-half to two-thirds sentenced to incarceration

In 2000, persons convicted of felony marijuana offenses were likely to be incarcerated. Half (51%) of the convictions for possession led to a prison or jail term, as did two-thirds (63%) of the trafficking convictions. Overall, one-third of all felony marijuana convictions resulted in a prison term of at least one year. This rate was the same for both marijuana trafficking and possession, raising questions regarding the charging phase of the proceedings that will be discussed later. As seen in Table 2, the distribution of persons sentenced to prison for trafficking and possession is similar, with the only dramatic departure in the use of probation and fines for persons sentenced for a marijuana trafficking felony.

**Table 2:** Felony State Sentences for Marijuana Convictions – 1990 and 2000

	**PRISON**		**JAIL**		**PROBATION**	
	**1990**	**2000**	**1990**	**2000**	**1990**	**2000**

**Possession**	30%	32%	22%	19%	48%	49%
**Trafficking**	31%	33%	42%	30%	27%	37%

Conventional wisdom suggests that individuals sentenced to prison for possession are repeat offenders with significant criminal histories. Although this may be true of many sentences, some states mandate incarceration even for some types of first-time marijuana possession. In Alabama, a 2004 report by the state's sentencing commission found that 328 people were sentenced to prison for marijuana possession, and only one-third were repeat offenders [60].

The rates for jail and probation sentences were relatively stable across time, although the proportion of persons sentenced to jail for marijuana trafficking dropped by nearly one-third while the proportion sentenced to probation increased by 37%.

#### • Average sentence length upwards of two years

Recent data on sentence length indicates that persons sentenced for a marijuana felony are likely to face sentences in the range of the national average for aggravated assault. In 2000, the average sentence for a person convicted of aggravated assault in a state court and sentenced to incarceration (prison or jail) was 37 months (median = 16 months), while the average sentence for persons sentenced to probation for a felony was 40 months (median = 36 months) [61]. An analysis of those figures for persons sentenced for marijuana felonies indicate a similar sentencing range. The average sentence for persons convicted of a marijuana felony in state court in 2000 was 28 months (median = 12 months) for incarceration and 40 months (median = 36 months) for probation [62].

Separated by type of marijuana offense, we find that possession cases actually result in more severe sentences than trafficking. Persons sentenced for trafficking received an average of 27 months (median = 9 months) incarceration, while those sentenced to probation received 39 months (median = 36 months). For possession, the incarceration average was 31 months (median = 16 months) and the probation average was 42 months (median = 36 months).

A potential explanation for marijuana possession cases receiving longer sentences, in general, than convictions for sale is due to the inclusion of "possession with intent to distribute" cases [63]. Whereas trafficking convictions require face-to-face purchases (frequently multiple purchases) by law enforcement in order to build a case of a criminal drug enterprise, possession with intent to distribute cases are based solely on the quantity of drugs found at the time of arrest. For example, if law enforcement agents exercise a warrant and enter a home to find a significant quantity of marijuana (more than deemed reflective of personal use) coupled with paraphernalia indicative of marijuana distribution, this evidence will likely be sufficient to merit a prosecutor pursuing a charge of possession with intent to distribute. Since the agents do not have evidence of a continuing criminal enterprise, a prosecutor is unlikely to seek a trafficking charge, which is more burdensome to prove. Because most trafficking cases are comprised of multiple purchases, any single charge for possession with intent to distribute will potentially elicit higher quantities of marijuana as well as the associated distribution paraphernalia than a typical series of face-to-face purchases by a law enforcement agent building a trafficking case.

The relatively stable patterns in state court conviction data, in light of the growth in arrests, raises questions about the allocation of law enforcement resources during the 1990s. While the numbers of arrests have more than doubled, making marijuana the single most pursued offense by American law enforcement agents, overall felony convictions have increased only modestly during the decade. Further, there is no discernable increase in the severity of marijuana offenses, since similar proportions are being sentenced to prison as in the past. Therefore, it appears that the court system is expending resources processing, dismissing, and filtering the increasing wave of marijuana arrestees. We estimate that 3.6% ($1.36 billion) of the national judicial and legal budget for 2001 ($37.8 billion) was spent on the court processing of marijuana offenders [64]. The fact that the upward trend in arrests is not reflected in felony conviction data suggests that the quality of arrests has diminished greatly. It is reasonable to surmise that the growth in marijuana arrests, which are primarily for possession, is laden with misdemeanor charges and cases that are dismissed by the prosecuting authority. From a policy standpoint, the question is whether this is an efficient use of law enforcement and court system resources.

### Marijuana in a city court system: a case study

There have been serious policy implications for criminal justice resources as a result of this shift in resources towards marijuana arrests. In New York City the increase in arrests due to the Giuliani-Bratton model of zero tolerance policing inundated the court system with marijuana cases. The number of arrests for marijuana increased 882% between 1990 and 2002, with an increase of 739% since Giuliani was elected in 1994, including a 1,877% increase in possession arrests [65]. This contributed to a backup of the court system to such a degree that by 1999 the number of overall cases dismissed because the deadline for a "speedy trial" had passed was up 20% from 1993 [66].

**Figure 9:**
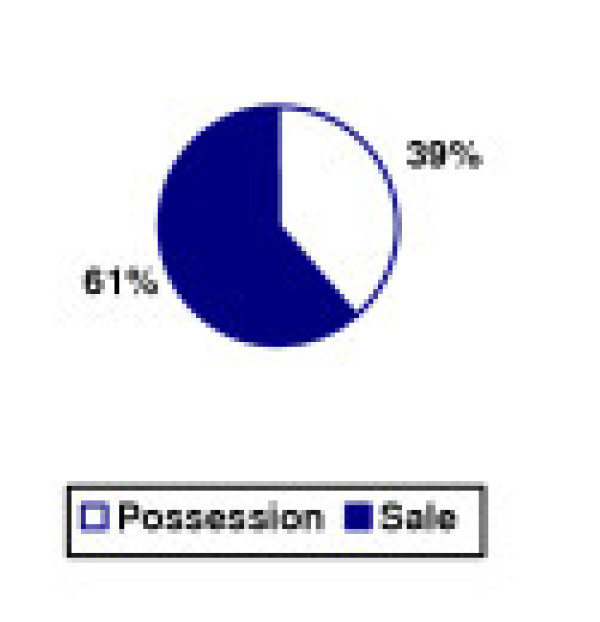
Marijuana Offense Dispositions by Type – New York City, 1994.

**Figure 10:**
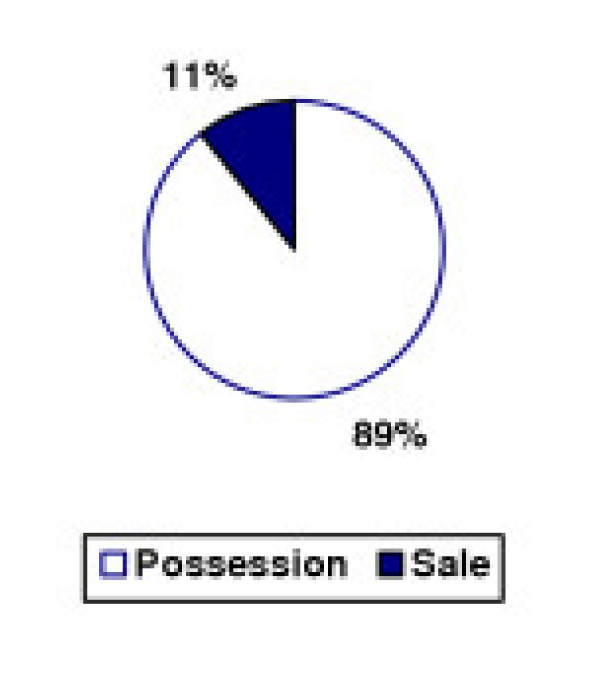
Marijuana Offense Dispositions by Type – New York City, 2002.

The disproportionate impact of possession arrests translates into court dispositions as well, as seen in Figures 9 and 10. In 1994, three of every five (61%) dispositions for marijuana in New York City courts were for sales offenses. By 2002, that figure had dropped to one of nine (11%). Overall, possession dispositions increased 1576% while conviction rates for sales dropped by one-third. All of the growth in marijuana possession dispositions was in the area of misdemeanor offenses. While lower court (misdemeanor) dispositions increased more than 17-fold, upper court (felony) dispositions for possession dropped 31%.

Meanwhile, the pattern in dispositions for sales offenses remained relatively flat. Lower court dispositions grew by 5% while the number of upper court dispositions was too low to arrive at any accurate statistical conclusions. The impact of zero tolerance policing on the judicial system is immediately evident upon examining New York City court data: a more than six-fold increase in marijuana dispositions, with a 15-fold increase in possession dispositions. The majority of this growth was from cases which the prosecution decided not to proceed with charging, indicated by a 23-fold increase in marijuana cases dismissed in lower court.

Although these cases did not require the level of resources accorded a full trial, there was a need to allocate court resources to address even those cases that were dismissed. Court staff time, processing costs, judge and attorney hours and other resources need to be dedicated even in cases that result in dismissal. This includes resources dedicated to arrest and processing by the police department followed by arraignment in the court system. By the time a case is dismissed, in addition to the arresting officer(s) and administrative staff necessary to process the defendant, the prosecutor's office and the office of the judge will also be involved. This comes at significant financial cost to the city as well as placing an additional burden upon an already stressed court system.

### Marijuana and the correctional system

The endpoint in the criminal justice system is corrections, where persons sentenced to supervision are either incarcerated in prison or jail, or in the community on probation or parole. Based on current prison population counts, we estimate that there are 27,900 persons in state and federal prison serving a sentence for which a marijuana violation is the controlling (or most serious) offense [67]. This translates to a national estimated loss of more than $600 million per year [68]. Twenty-three percent of marijuana offenders are incarcerated for a possession offense, 15% for possession with intent to distribute, and 59% for trafficking. Of the total, 40% are incarcerated for the first time, 48% are recidivists with no current or prior violent offense history, and 12% are recidivists with a past violent offense in their criminal history.

This initial analysis raises questions about the severity of offenders incarcerated in state and federal prisons for marijuana offenses. Nearly 90% have no history of a violent offense. However, violent offenses are not the only measure of a person's risk to society. Many officials assert that prison sentences for marijuana are imposed for high level offenses. In order to address this question, we analyzed the data from the *Survey of Inmates *on offender role in a drug enterprise.

The *Inmate Survey *asks respondents to report their activity in the drug trade. Although self-report data suffers from some inherent biases, it is a much better indicator of individual level drug involvement because, unlike the controlling offense, it is not impacted by pre-trial discretion and negotiations regarding charging level (as discussed in the section on court processes). We define drug activity as high-level if the individual has been involved in "importing," "manufacturing," "money laundering" or "distribution to other sellers." We estimate that 48% of marijuana offenders in state and federal prison were engaged in high-level drug activity prior to their arrest. Federal marijuana offenders participated in high-level activity at a higher rate (65%) than state prisoners (40%).

Using reported activity response as an indicator, there is reason to question the assertion that only serious marijuana distributors are incarcerated in prison. In fact, the data strongly indicate that a significant number of marijuana offenders are in prison for playing a low-level role in the drug market. We can see this by identifying only those persons in state or federal prison on a first-time offense, who had not played a role of importer, manufacturer, or distributor of marijuana, and who did not involve a weapon in their offense [69]. Table 5 illustrates that when these characteristics are taken into consideration, there are still 6,600 (24%) marijuana offenders in prison for a low-level offense. Based upon these criteria, we conclude that at least one in four persons in prison for a marijuana offense can be classified as a low-level offender.

**Table 5:** Marijuana Offenders in State and Federal Prison

**Total Marijuana Offenders in Prison**	***27,900 (100%)***
Total First-Time Marijuana Offenders in Prison	*11,200 (40%)*
No weapon	*10,400 (37%)*
No weapon, No importation	*8,500 (30%)*
No weapon, No importation, No manufacturing	*8,700 (30%)*
No weapon, No importation, No manufacturing, No laundering	*8,300 (30%)*
No weapon, No importation, No manufacturing, No laundering, No distribution	*6,600 (24%)*

In addition to persons serving a sentence in state or federal prison for a marijuana offense, there are a greater number of people on probation or parole or in jail. Although data for these populations is not available at the same level of detail as for persons in prison, we were able to create estimates of the number of people on probation or sentenced to jail for a marijuana offense using data from the *National Judicial Reporting Program*. Based on sentencing patterns in 2000, we estimate in 2003 that 36,000 people were on probation for a marijuana offense and an additional 4,600 were in jail serving a sentence for marijuana. These jail numbers do not include pre-trial detainees awaiting court proceedings. Thus, with half of the nearly 700,000 persons in jail awaiting trial, we estimate that the number of those persons who have been charged with a marijuana offense will equal or exceed the 4,600 people that have been sentenced.

Based on the available data for prison, jail, and probation, we estimate that over 68,000 people are under correctional supervision for a marijuana offense. While there are no data regarding the proportion of persons on parole for a marijuana offense, it is likely that this group would raise the total number of persons under supervision to more than 75,000. In addition, there are an unknown number of persons in prison due to a probation or parole violation for a non-marijuana offense who have had their supervision revoked after testing positive for marijuana.

## Discussion and recommendations

It is apparent that despite a rapidly evolving national dialogue around marijuana use and a renewed discussion of alternatives to arrest and incarceration, during the 1990s the law enforcement community pursued marijuana offenses with a renewed vigor. Arrests for possession came to dominate nearly all of the growth in drug arrests during the period studied. Assertions that "nobody" goes to prison for marijuana are misguided and over-simplify the policy issue. Modest numbers of persons serving time in prison for a marijuana offense does not necessarily mean that the country is effectively calibrating its resources to address marijuana use.

Narrowly focusing on people incarcerated in state and federal prison for marijuana offenses diverts the lens of analysis from the real target: low-level marijuana users. These persons have disproportionately been targeted by the war on drugs in the 1990s. Increased arrests and frequent use of probation and suspended sentences may give the appearance that the correctional system has been calibrated properly to only incarcerate the most severe offenders, but a discussion of resource allocation demands that we also consider the growth of persons with an arrest and felony conviction record as a result of this policy. Such persons face many of the same challenges and obstacles as people who have been incarcerated. These include a denial of federal financial aid for higher education, lack of access to federal aid such as food stamps, denial of entry to public housing, and a prohibition on the right to vote, in some states for life. In addition to the institutional hurdles, there remain informal barriers for persons with a felony conviction, such as the difficulty to compete for employment with a criminal record. All of these critical issues are a cost of the drug war and exist equally whether one spends time in prison or serves a sentence in the community.

Moreover, there are important policy questions regarding the growth of marijuana arrests and the impact on law enforcement and court processing resources. As states continue to struggle under budgetary constraints, the wisdom of making nearly 700,000 marijuana arrests annually, the majority of which will be dismissed or processed as misdemeanors, is called into question. Proponents of public order, or "broken windows" policing, maintain that these arrests are symbolic and serve to maintain order, which leads to the suppression of more dangerous crime. However, this is a contentious point, and more than twenty years after the philosophy was put forth by James Q. Wilson and George Kelling, there remains no empirical validation of its truth. Criminologist Ralph Taylor notes that "initial incivilities contribute to later changes in some serious crimes ... [b]ut the contributions are neither as sizable as anticipated, nor as consistent ... [70].

What is empirically evident is that the growth in marijuana arrests over the 1990s has not led to a decrease in use or availability, nor an increase in cost. Meanwhile, billions are being spent nationally on the apprehension and processing of marijuana arrestees with no demonstrable impact on the use of marijuana itself, or any general reduction in other criminal behavior. Our analysis of criminal justice processing of marijuana use over the 1990s suggests that the contemporary approach is apportioning resources inefficiently at each stage of the system. In order to address issues of marijuana and the criminal justice system in a more effective manner, policymakers and practitioners should consider the following recommendations.

### Law enforcement

#### Prioritize arrest policies

As has become policy in jurisdictions such as Seattle and Oakland, law enforcement agencies should categorize enforcement of marijuana possession as a low priority so as to conserve police resources for more serious offenses.

#### Eliminate marijuana enforcement as a means of "broken windows" policing

Marijuana arrests in some cities have been justified on the premise that arresting people for marijuana possession disrupts other, potentially more serious, behaviors. Such strategies result in substantially increased numbers of low-level marijuana arrests, with little evidence that they are actually effective in suppressing other criminal behaviors. Further, they contribute to the mistrust of law enforcement, particularly in communities of color that have been disproportionately targeted by such practices.

### Courts

#### Exercise prosecutorial discretion to divert cases from the court system

Few marijuana possession arrests result in any significant jail or prison time, yet they are cumulatively quite costly to the court system through the engagement of prosecutors, defense counsel, judges, and probation officers. Prosecutors should use their discretion in appropriate cases to drop charges and/or utilize community resources at the earliest possible stage of court proceedings in order to effect outcomes that represent a reasonable allocation of resources.

#### Exercise prosecutorial discretion to reduce the number of felony convictions

In most states felony drug convictions carry a set of collateral consequences in addition to whatever punishment is directly imposed. These may include a ban on receipt of welfare benefits, prohibition on living in public housing, loss of student loans, and loss of the right to vote. These punishments place additional burdens on ex-offenders attempting to reenter the community. Therefore, to the extent that the interests of justice can be served through a misdemeanor conviction rather than a felony, prosecutors should use their charging discretion to pursue such outcomes.

### Policy

#### Encourage debate on marijuana policy

National debate on drug issues has too often been characterized by "soundbites" that distort the policy issues under consideration. In the case of marijuana, proposals for decriminalization represent an alternative approach to current policy. Consideration of such options should be addressed in the context of the findings of this report, including the substantial criminal justice and social costs involved in the large-scale prosecution of marijuana offenders. National debate on marijuana policy, and drug policy generally, should be focused on the most effective ways of addressing substance abuse and the most efficient allocation of law enforcement resources.

#### Federal government should respect local decisions

For the period of the war on drugs, federal funding – currently $19 billion a year – has been allocated in a 2:1 ratio of enforcement to treatment/prevention [71]. These priorities have resulted in a bloated prison population, with high proportions of low-level offenders. The Federal government should defer to local governments to develop their own approaches to marijuana use and respect the choices of state, county, and city policymakers. Federal funding should not be tied to a locality's decision to address marijuana use in only one fashion, namely law enforcement; rather, it should also encourage and adequately fund alternative strategies. A number of cities have raised concerns about the emphatic prosecution of marijuana as putting undue stress upon law enforcement resources, culminating in calls for and implementations of policy changes. The federal government should recognize these developments, and respect the choices of communities and local government agencies.

**Figure 2:**
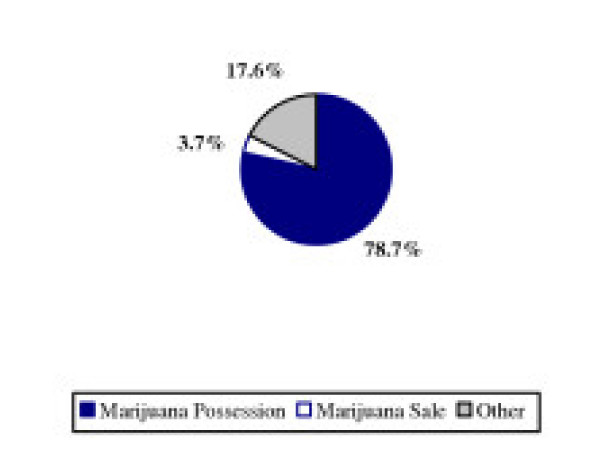
Marijuana as a Proportion of Growth in Drug Arrests – 1990 to 2002.

**Figure 6:**
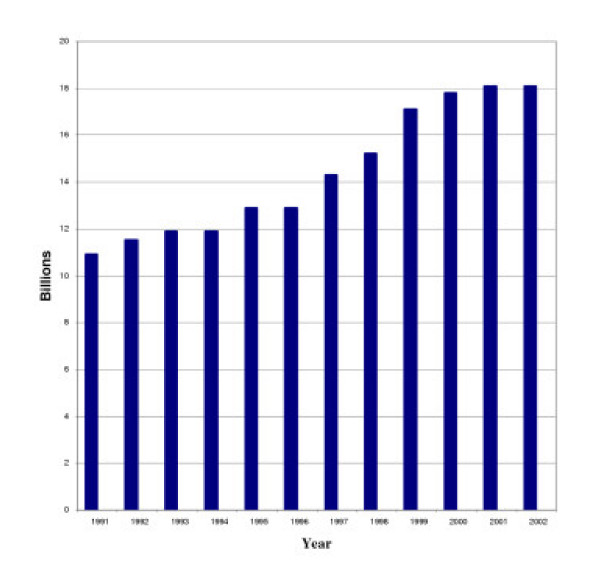
Federal Drug Control Budget – 1991 to 2002**. ** = Chart adapted from Pastore and Maguire, Table 1.12.

**Figure 8:**
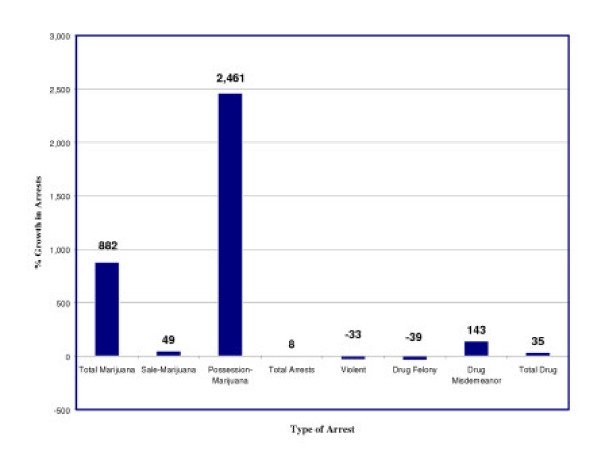
Growth in Arrests in New York City – 1990 to 2002.

**Table 3:** Average Felony Sentence (months) in State Court – 2000

	**All Offenses**	**Aggravated Assault**	**Marijuana**	**Marijuana Possession**	**Marijuana Trafficking**
***Prison/Jail***	36 (16)*	37 (16)	28 (12)	31 (16)	27 (9)
***Probation***	38 (36)	40 (36)	40 (36)	42 (36)	39 (36)

*Median in parentheses

**Table 4:** Criminal History of Marijuana Offenders

**First-Timers**	**Recidivist/Non-Violent**	**Recidivist/Violent**
40%	48%	12%
